# Role of tumor necrosis factor-alpha in the central nervous system: a focus on autoimmune disorders

**DOI:** 10.3389/fimmu.2023.1213448

**Published:** 2023-07-07

**Authors:** Natalia Gonzalez Caldito

**Affiliations:** Department of Neurology, Northwestern Memorial Hospital, Feinberg School of Medicine, Northwestern University, Chicago, IL, United States

**Keywords:** TNF-a, cytokines, central nervous system, neuroinflammation, multiple sclerosis, neurosarcoidosis, Neuro-Behcet’s disease, microglia

## Abstract

Tumor necrosis factor-alpha (TNF-α) is a pleiotropic immune cytokine that belongs to the TNF superfamily of receptor ligands. The cytokine exists as either a transmembrane or a soluble molecule, and targets two distinct receptors, TNF-α receptor 1 (TNFR1) and TNF-α receptor 2 (TNFR2), which activate different signaling cascades and downstream genes. TNF-α cellular responses depend on its molecular form, targeted receptor, and concentration levels. TNF-α plays a multifaceted role in normal physiology that is highly relevant to human health and disease. In the central nervous system (CNS), this cytokine regulates homeostatic functions, such as neurogenesis, myelination, blood-brain barrier permeability and synaptic plasticity. However, it can also potentiate neuronal excitotoxicity and CNS inflammation. The pleiotropism of TNF-α and its various roles in the CNS, whether homeostatic or deleterious, only emphasizes the functional complexity of this cytokine. Anti-TNF-α therapy has demonstrated effectiveness in treating various autoimmune inflammatory diseases and has emerged as a significant treatment option for CNS autoimmune diseases. Nevertheless, it is crucial to recognize that the effects of this therapeutic target are diverse and complex. Contrary to initial expectations, anti-TNF-α therapy has been found to have detrimental effects in multiple sclerosis. This article focuses on describing the various roles, both physiological and pathological, of TNF-α in the CNS. Additionally, it discusses the specific disease processes that are dependent or regulated by TNF-α and the rationale of its use as a therapeutic target.

## Introduction

Tumor necrosis factor-alpha (TNF-α) is a pleiotropic immune cytokine belonging to the TNF superfamily of receptor ligands. It is involved in several homeostatic and inflammatory processes, and plays a major role in autoimmune and inflammatory diseases (1–3). Although it is crucial for the normal immune response, its inappropriate production can be deleterious (1). TNF-α is a key regulator of acute and chronic inflammation and, in certain circumstances, causes cell death by apoptosis and necroptosis (2, 4–6). The diverse effects of this cytokine depend on its receptors, TNF receptor 1 (TNFR1) and TNF receptor 2 (TNFR2), and their distinct downstream signaling pathways (7).

TNF-α is one of the main cytokines regulating inflammation in the human body (6). It is produced primarily by cells of the innate immune system, such as macrophages and natural killer (NK) cells, as well as cells of the adaptive immune system, specifically, activated T cells (8, 9). In addition to its involvement in systemic inflammation, TNF-α plays important physiopathological roles in the central nervous system (CNS) (3, 9, 10). At homeostatic levels, TNF-α is involved in neuroplasticity and myelination although, in pathological levels it might cause excitotoxicity, neuroinflammation and breakdown of the blood-brain barrier (11).

A number of studies have implicated TNF-α in autoimmune inflammatory diseases of the CNS (12). It plays a critical role in the pathogenesis of several disorders, and pharmacologically targeting of TNF-α has shown clinical benefit (2, 13). For instance, anti-TNF-α therapy has been successfully used in the treatment of neurosarcoidosis and neuro-Behçet’s disease. However, the role of TNF-α is complex and pleiotropic, and its blockade can result in unintended biological effects as well (14). In multiple sclerosis (MS), TNF-α blockade can lead to paradoxical worsening of the disease (15). Furthermore, central and peripheral demyelination can be observed with anti-TNF-α therapy, even in rheumatological disorders, which are not primarily categorized as neurological diseases (16, 17).

In this review, we discuss the role of TNF-α in the pathogenesis of the main autoimmune disorders of the CNS and its significance as a therapeutic target.

## TNF-α and receptor signaling

TNF-α exists as a transmembrane and a soluble molecule, and targets two natural receptors, TNFR1 and TNFR2 (4). Transmembrane TNF-α (tmTNF-α) is processed by TNF-α converting enzyme (TACE, aka ADAM17) into soluble TNF-α (sTNF-α). Additionally, sTNF-α can exists in two different forms, trimeric or active form and monomeric or inactive form. The cytokine receptors exhibit differential cellular expression and elicit independent signaling responses (1, 18). TNFR1 is ubiquitously expressed in all cells and can be activated by both tmTNF, and sTNF. In contrast, TNFR2 is activated by tmTNF and is present only in certain cell types such as immune cells (myeloid cells, specific B and T cell subsets), endothelial cells, cardiomyocytes and neurons (2, 19). Furthermore, TNFR2 has been shown to be overexpressed in cancer cells and to promote proliferation and tumor growth. In fact, this receptor is emerging as a therapeutic target in cancer therapy (20, 21).

**Figure 1:**
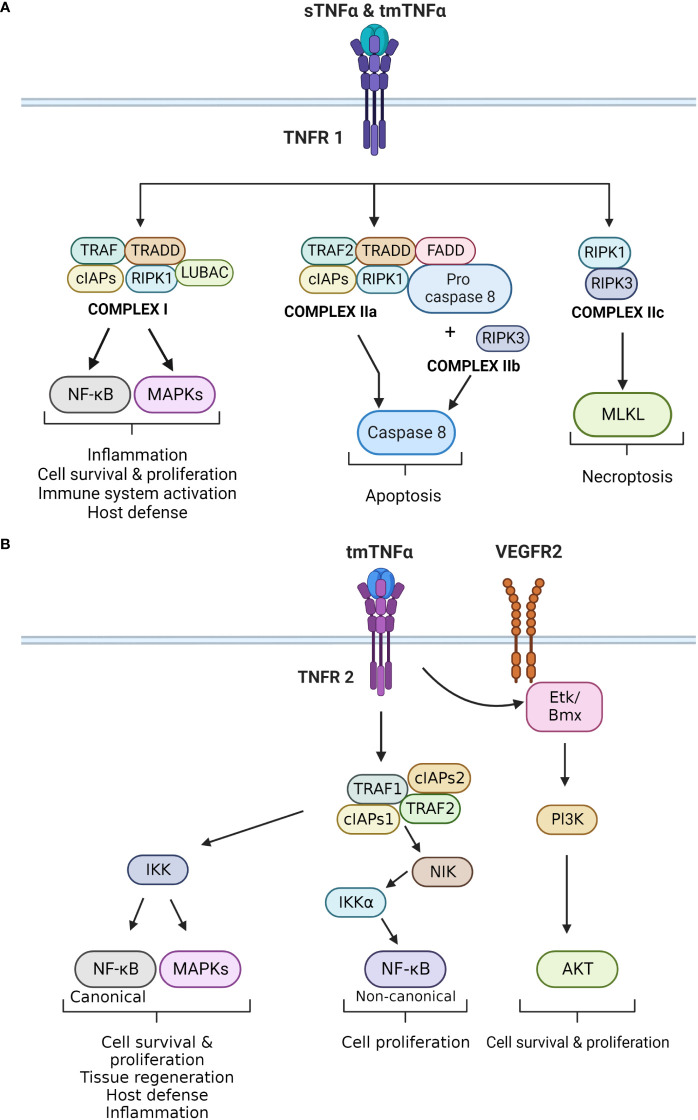
**(A)** Principal signaling pathways of TNFR1. The different effects of TNF-α upon binding to TNFR1 are displayed. TNFR1 can bind to sTNF-α and tmTNF-α. It forms four different complexes, which exert different cellular effects. Complex I is mainly involved in inflammation, cell survival and proliferation by activating the canonical NF-kB and MAPKs pathways. Complex IIa and IIb activate caspase 8 that leads to apoptosis, whereas complex IIc activates MLKL causing necroptosis. **(B)** Principal signaling pathways of TNFR2. The different effects that result in activation of TNFR2 are demonstrated in this figure. TNFR2 is activated by tmTNF-α, which leads to the formation of the TNFR2 complex. This complex exerts two main downstream effects. The first results in the activation of IKK, which triggers the canonical NF-kB and MAPKs pathways. Both impact cell survival and proliferation, as well as inflammation and host defense. The second effect is the activation of NIK, which phosphorylates IKKα. This kinase activates the non-canonical NF-kB pathway, which also regulates cell proliferation. In addition, TNFR2 can induce VEGFR2 signaling in the absence of a ligand. TNFR2 can activate ETk/Bmx, and ultimately the Pl3K/AKT pathway that regulates cell survival and proliferation.

Activation of TNFR1 by TNF-α triggers the formation of four distinctive molecular complexes (**Figure 1A**). Complex 1 is formed in the cell membrane, while complex IIa, IIb and IIc are formed in the cytoplasm. The formation of complex 1 is initiated by the receptor binding to TNFR1 associated death domain (TRADD) adaptor protein, followed by the recruitment of signaling proteins including receptor-interacting serine/threonine-protein kinase 1 (RIPK1), TNFR-associated factor 2 or 5 (TRAF2/5), cellular inhibitor of apoptosis protein 1 or 2 (cIAP1/2), and the linear ubiquitin chain assembly complex (LUBAC). Once formed, the complex targets IκB kinase (IKK), which activates nuclear factor κB (NF-κB) and members of mitogen-activated protein kinases (MAPKs) family, such as c-JUN kinase (JNK) and p38 MAPK (1, 4, 22). These signaling pathways regulate cell survival and proliferation, as well as immune activation and inflammation. NF-κB is a major regulatory transcription factor that controls the expression of several pro-inflammatory cytokine genes (14). Specifically, NF-κB activation and translocation into the cell nucleus induce the transcription of IL-6, IL-1β and TNF-α itself. Together, TNF-α and NF-κB form a positive activation/expression loop that facilitates and amplifies the inflammatory responses.

Complex IIa and IIb are structurally and functionally compatible as both lead to caspase-8-dependent apoptosis. Complex IIa is formed by TRADD, RIPK1, TRAF2, cIAP1/2, pro-Caspase-8, and Fas-associated protein with death domain (FADD). Complex IIb is similar to IIa, but also contains RIPK3. The formation of this complex is independently regulated by the long isoform of FLICE-like inhibitory protein (FLIP_L_), which has a dual role in apoptosis and caspase activation (4). The last complex assembled by TNFR1 is complex IIc and it is formed by RIPK1 and RIPK3. This complex activates the mixed lineage kinase domain-like protein (MLKL) and triggers necroptosis, a pro-inflammatory form of cell death. Necroptosis differs from apoptosis, as it causes disruption of the plasma membrane and release of intracellular contents into the extracellular space, leading to a local inflammatory response (2).

On the other hand, TNFR2 is primarily activated by tmTNF-α through a cell-to-cell interaction (**Figure 1B**). It lacks the dead domain FADD, thus is unable to trigger directly trigger cell death (22). TNFR2 exerts different effects depending on the pathways that are activated (19). TNFR2 forms a complex with TRAF2, TRAF1, cIAP1 and cIAP2. This complex interacts with NF-κB-inducing kinase (NIK), leading to phosphorylation of IKKα, and activation of the non-canonical NF-κB pathway (22, 23). Additionally, TNFR2 can phosphorylate endothelial/epithelial protein tyrosine kinase (Etk/BMX), which is typically associated the vascular endothelial growth factor receptor 2 (VEGFR2). This phosphorylation results in cross-activation of the PI3K/Akt pathway, enhancing cells survival and proligeration (22).

TNFR2 primarily participates in homeostatic activities such as cell proliferation, survival, and tissue regeneration. It exhibits both pro- and anti-inflammatory effects, and plays a significant role in regulating and attenuating inflammatory responses (19). Importantly, TNFR2 has a protective role against autoimmunity by promoting and enhancing the activity of T regulatory cells (24, 25). Furthermore, TNFR2 stimulates T effector cells to produce IL-2, which inhibits the differentiation of Th17 cells. TNFR2 also neutralizes tmTNF-α by shedding it into sTNF-α, thereby inhibiting IL-6 secretion (26).

In addition to its role in cell survival and proliferation, TNFR2 can also induce cell apoptosis (22, 27). This occurs through crosstalk between TNFR1 and TNFR2, as they share common molecules in their signaling pathways, such as TRAFs and c-IAPs (27). TNFR2 activation leads to the depletion of these molecules, resulting in different cellular effects (22). For example, it can inhibit the formation of TNFR1 complex I, which activates the MAPK and NF-κB pathways involved in cell survival. On the other hand, TRAF2–c-IAP1/2 complex can interfere with caspase 8 activation, leading to the formation of the necrosome or complex IIc (22).

## Homeostatic effects of TNF-α in the CNS

**Figure 2:**
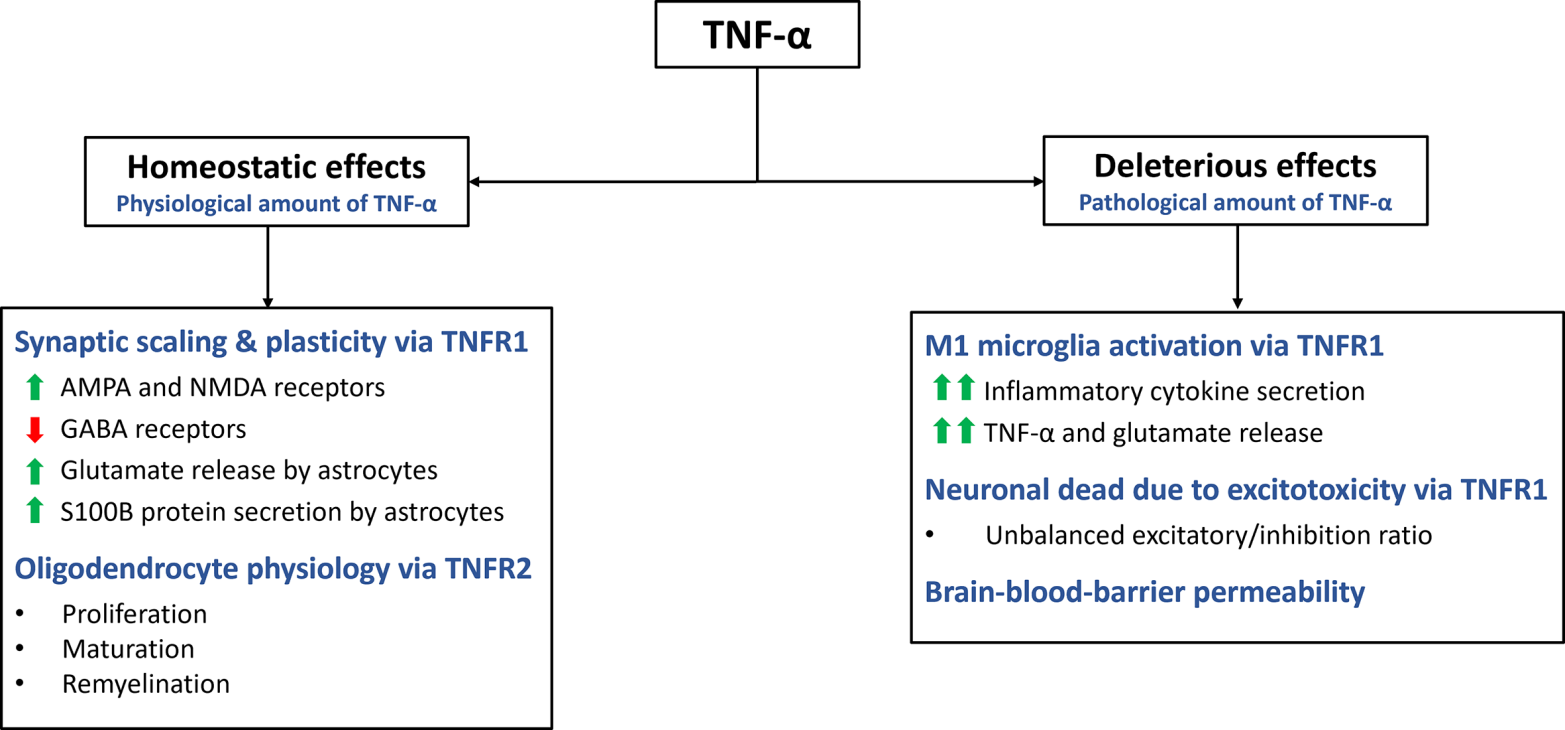
Homeostatic and deleterious effects of TNF-α in the CNS. The main effects of TNF-α are summarized in this figure. TNF-α plays a role in various homeostatic functions. It is involved in synaptic plasticity and scaling, exerting excitatory effects on the synapse by increasing AMPA and NMDA receptors while decreasing GABA receptors. Additionally, it stimulates astrocytes to secrete S100B, which helps maintain calcium and zinc levels in the synapse. Through TNFR2 activation, TNF-α promotes oligodendrocyte proliferation, maturation, and remyelination. However, TNF-α can also have deleterious effects through TNFR1. It activates microglia, leading to a pro-inflammatory M1 profile characterized by cytokine secretion and further release of TNF-α and glutamate. Excessive TNF-α levels disrupt the excitatory/inhibitory balance, resulting in excitotoxicity. TNF-α also increases the permeability of the blood-brain barrier.

Apart from its role in inflammation, cell death and proliferation, TNF-α also plays a critical role in maintaining CNS homeostasis (**Figure 2**) (1, 14). It is expressed by various cells types in the CNS, including glia cells, microglia, astrocytes, and neurons (11). TNF-α contributes to multiple aspects of CNS function, such as synaptic homeostasis, transmission and scaling. It also modulates processes such as excitotoxicity, neuroinflammation and blood-brain barrier permeability (14). Additionally, TNF-α plays a pivotal role in regulating oligodendrocyte cell survival, myelin formation and repair (28).

TNF-α plays a crucial role in the synaptic formation and function, contributing to neuroplasticity and synaptic scaling (29). Synaptic scaling refers to the adjustment of synaptic strength in response to prolonged changes in electrical activity (30). TNF-α influences both presynaptic and postsynaptic activity in neurons, including the expression of neurotransmitter receptors (9, 31). Glutamate, the primary excitatory neurotransmitter in the CNS, binds to amino-3-hydroxy-5-methyl-4-isoxazolepropionic acid (AMPA) receptors (AMPARs) to mediate excitatory signals. Modulation of AMPARs expression in postsynaptic neurons is crucial for synaptic neuromodulation. TNFR1, enhances the activity of excitatory synapses by upregulating the surface expression of AMPARs in neurons (30, 32). This upregulation of AMPARs is believed to contribute to the strengthening of synaptic transmission. Additionally, TNF-α has been found to upregulate the expression of N-methyl-D-aspartate receptors (NMDARs), through a similar mechanism (11, 32). This suggests that TNF-α can impact both AMPAR and NMDAR activity, further influencing synaptic function and plasticity.

In contrast, TNF-α downregulates the surface expression of gamma-aminobutyric acid A receptors (GABAARs). GABAAR mediate inhibitory neurotransmission and their downregulation leads to a reduction in the activity of the inhibitory synapses (11). By altering the balance between excitation and inhibition, TNF-α acts as a neuromodulatory cytokine that can increase the excitatory/inhibitory ratio in postsynaptic neurons (30). This modulation of synaptic activity by TNF-α highlights its role in shaping synaptic plasticity and the overall excitability of neural circuits.

TNF-α exerts various effects on the CNS beyond its influence on receptor expression in the synaptic cleft. One notable effect is its ability to modulate the release of glutamate from astrocytes, altering the local concentration of this excitatory neurotransmitter (33). TNF-α stimulates the secretion of S100B, a zinc-binding protein, by astrocytes, which plays a role in maintaining calcium and zinc levels in the synapse. By regulating zinc metabolism and co-releasing mechanisms of zinc and glutamate, TNF-α indirectly modulates excitatory synaptic transmission (11, 34). However, it is important to note that high concentrations of TNF-α can disrupt zinc levels, leading to reduced synaptic activity and potential cognitive impairment (9).

Furthermore, TNF-α plays a role in the regulation of oligodendrocyte proliferation and myelin formation, although the precise mechanisms are not fully understood (28, 35). TNF-α, through its interaction with TNFR2, promotes the proliferation of oligodendrocyte progenitor cells and supports the maturation of oligodendrocytes, which are essential for myelin production (28). However, the effects of TNF-α on oligodendrocytes are complex and multifaceted. Astrocytes can cause TNF-α-induced toxicity to oligodendrocyte progenitor cells, suggesting a potential detrimental role in certain contextx (35). It is well known that TNF-α has proinflammatory effects through TNFR1, while also contributing to myelination through TNFR2 (36). Further investigation is needed to fully understand the underlying mechanisms and the functional implications of TNF-α’s actions on oligodendrocytes in the context of CNS homeostasis and disease processes.

## TNF-α, excitotoxicity and neuroinflammation

TNF-α plays a crucial role in the induction and augmentation of inflammation within the CNS (3). It is primarily produced by activated microglia, but it can also be synthesized by the endothelial cells, neurons and infiltrating immune cells. The local production of TNF-α by glial cells can be upregulated in response to other inflammatory cytokines that are delivered to the CNS by infiltrating T cells. For example, interferon-gamma (IFN-γ), stimulates microglia to produce TNF-α, leading to further increases in its local levels (37). The coordinated actions of TNF-α and IFN-γ contribute to the promotion of oxidative stress, cell injury and tissue damage, ultimately driving the inflammatory response in the CNS.

One of the key functions of TNF-α in CNS is its involvement in the regulation of excitotoxicity and neuroinflammation (**Figure 2**) (18). These two processes are closely interconnected, largely due to the multifaceted effects of TNF-α. The cascade of events associated with excitotoxicity, ultimately leads to neuronal cell death, triggering local neuroinflammation (11). TNF-α plays a pivotal role in each step of this pathological process:

1) TNF-α contributes to excitotoxic cell death in neurons by altering the balance between excitatory and inhibitory signals, leading to an increased influx of calcium into the cells (11) (38). This neuronal death, in turn, initiates a local inflammatory response, upregulates TNF-α expression, and establishes a positive feedback loop.
2) TNF-α also regulates the release and re-uptake of glutamate in astrocytes (39). Moreover, glutamate stimulates microglial production of TNF-α through its interaction with mGlutR2 receptors.
3) Activation of microglial cells and their induction to the proinflammatory M1 phenotype are medicated by TNF-α (11, 40, 41). The M1 microglial phenotype, characterized by increased expression of proinflammatory cytokines such as IL-1β, IFN-γ, IL-6, IL-8 and IL-12 and other proinflammatory genes is dependent on the NF-κB signaling pathway (10). Conversely, the alternative M2 microglial phenotype, induced by IL-4, IL-10 and IL-13 and TGF-β, exerts anti-inflammatory and neurotrophic effects (10).
4) TNF-α plays a crucial role in the disruption of the blood-brain barrier and the infiltration of inflammatory cells and molecules into the CNS. This process, medicated by the TNFR1,is associated with increased expression of adhesion molecules (VCAM and ICAM) by endothelial cells, cell injury (necroptosis) and compromised barrier function (42, 43).

## Role of TNF-α autoimmune diseases of the CNS

Neuroinflammation is a common pathological characteristic of several CNS disorders. It is characterized by the activation of glial cells, demyelination and neurodegeneration (44). In autoimmune diseases of the CNS, TNF-α is a key inflammatory cytokine that regulates peripheral and local immune responses. Its involvement in excitotoxicity and neuroinflammation highlights its impact on CNS pathophysiology and emphasizes its multifaceted roles in regulating cellular responses and inflammatory processes within the CNS.

## Neurosarcoidosis

Sarcoidosis is a multisystemic inflammatory disease of unknown etiology characterized by granuloma formation in the affected organs. It can involve the CNS as a primary disorder (neurosarcoidosis) in 10-20% of all cases or as a secondary disorder in 5-20% of all systemic cases (13, 45). Neurological presentations are diverse (46), but certain manifestations such as cranial neuropathies, meningitis or myelopathy are most typical in this disease (13). Its epidemiology suggests a complex multifactorial disease process involving interactions between environmental factors and genetic predisposition (47, 48). It is thought to be autoimmune disorder where an unknown antigen triggers the activation of the innate immune system, resulting in a delayed type of hypersensitivity (DTH) reaction and granuloma formation (13).

**Figure 3:**
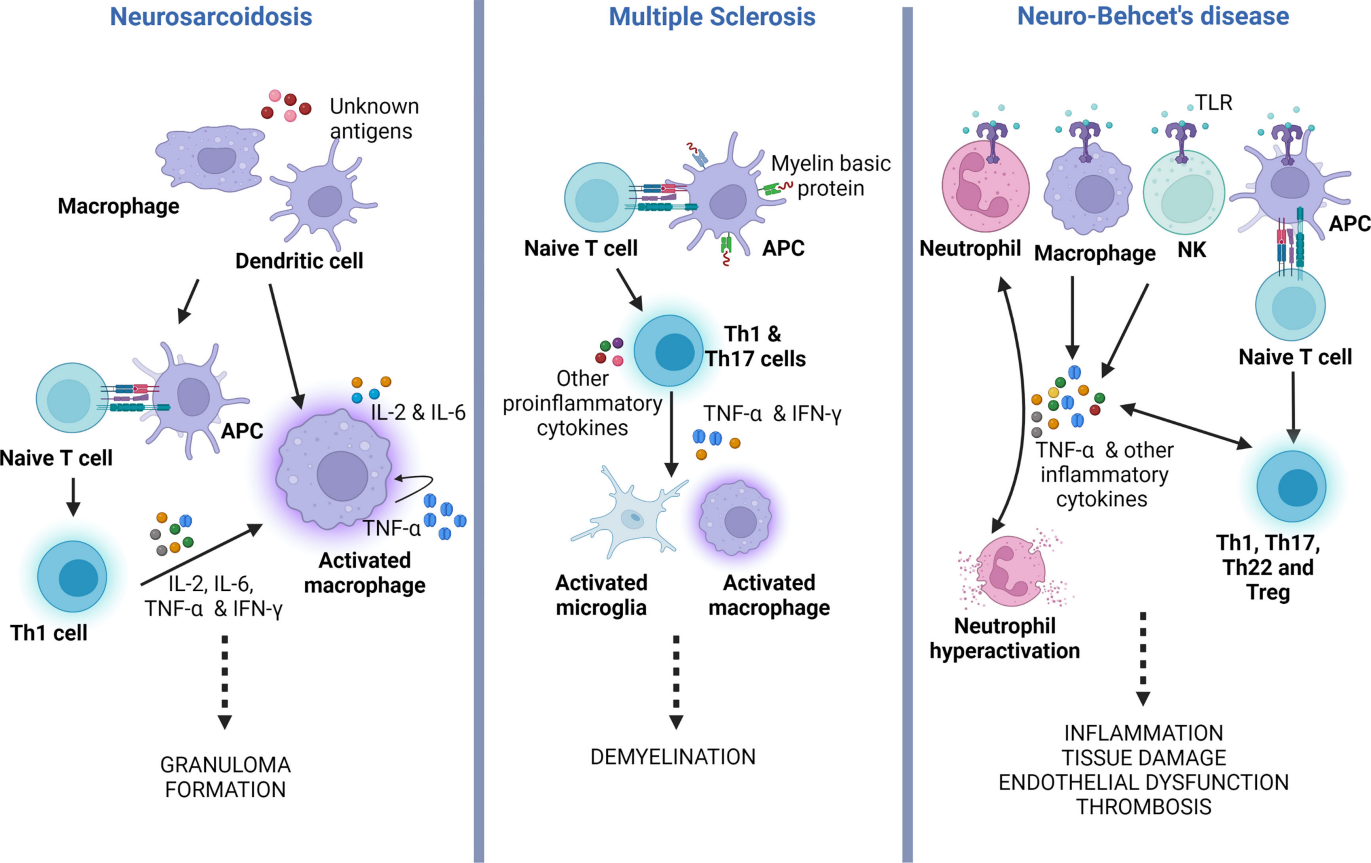
TNF-α role in the pathogenesis of neurosarcoidosis, multiple sclerosis and neuro-Behçet’s disease. Simplified illustration displaying the main roles of TNF-α in the physiopathology of neurosarcoidosis, MS and neuro-Behçet’s. In neurosarcoidosis, TNF-α is produced by macrophages, which further stimulates the generation of TNF-α, forming a positive feedback mechanism. It also induces T naïve (Th0) cells to polarize to Th1 phenotype that facilitates macrophage activation and granuloma formation. In MS, TNF-α secreted by Th1 and Th17, which results in activation of microglia and macrophages, both potentiating CNS demyelination. In neuro-Behçet’s disease, TNF-α is produced by different immune cells that contribute to neutrophil activation and generation of other pro-inflammatory cytokines, all causing chronic inflammation and multiorgan tissue damage. APC, antigen presenting cell; TLR, toll-like receptor; Treg, T regulatory cell; Th, T helper cell; NK, natural killer cell; IFN-γ, Interferon-gamma; TNF-α, tumor necrosis factor alpha.

The unifying pathological findings in sarcoidosis are non-caseating granulomas. These granulomas consist of multinucleated giant cells and epithelioid macrophages surrounded by a rim of lymphocytes, mainly CD4 T cells and occasional B and CD8 T cells (49, 50). The initiation of granuloma formation involves macrophages and dendritic cells, which acts as antigen presenting cells (APCs) (49). Activated macrophages release proinflammatory cytokines (IL-1, IL-12, TNF-α), biasing the antigen presentation towards a delayed hypersensitivity (DTH) response (**Figure 3**, left panel). TNF-α is involved in the activation and differentiation of the Th0 into Th1 cells, a process dependent on NF-kB and MAPK signaling pathways. Subsequently, Th1 cells secrete other cytokines such as IL-2, IL-6 and IFN-γ, which promote DTH, accelerate macrophage activation, cellular migration towards the site of inflammation and tissue infiltration (47, 51) TNF-α also stimulates the differentiation of T regulatory cells (Treg) and NK cells, amplifying the cellular immune response and granuloma formation (52).

The essential role of TNF-α in granuloma formation is further supported by the clinical efficacy of anti-TNF-α therapies. These therapies have demonstrated effectiveness in controlling neurosarcoidosis, decreasing lesion size and activity, and improving treatment outcomes (53). Infliximab and adalimumab, the main drugs of this class, are used in clinical practice, as a first- and second-line of therapies respectively (54). However, it is important to note that anti-TNF therapies can paradoxically lead to sarcoidosis-like reactions, which may require discontinuation of the treatment (51, 53, 55). Although these reactions are rare, they are thought to be associated with the stability of the drug-target complexes (55). Infliximab and adalimumab form relatively stable complexes with TNF-α and fix complement, which facilitates their clearance (56). On the other hand, etanercept, which is more commonly linked to sarcoidosis-like reactions, forms less stable complexes that can dissociate and allow the release of TNF-α. However, the exact mechanism underlying these reactions remains not completely understood, as the pharmacodynamics and pharmacokinetics of the different anti-TNF-α therapies only partially explain their occurrence (57).

## Multiple sclerosis

Multiple sclerosis (MS) is a chronic inflammatory demyelinating disease of the CNS (58). It is relatively common and it is thought to have a worldwide prevalence of 2.3 million (59). MS is a heterogeneous disease that can cause a wide spectrum of neurological symptoms. The severity of these symptoms can vary depending on the localization and extent involvement of the CNS (60). The etiology of MS remains unknown, but it is believed to be an autoimmune disorder influenced by multiple risk factors, including genetics, environment and infections (61, 62). Inflammatory demyelinating lesions, which are characteristic of the disease, typically occur around postcapillary venules in areas of the CNS that contain myelin. Pathological studies of MS have revealed multifocal aggregates of infiltrating T cells, B cells, macrophages and activated microglia. Tissue damage is characterized by demyelination, astrocytosis, and injury to neurons and oligodendrocytes (63).

CNS inflammation in MS is a complex and multifocal process involving the interactions between the innate and adaptive immune systems. Autoreactive T cells are thought to arise in the peripheral immune system when immune tolerance fails, leading to the presentation of myelin-mimicking protein epitopes (64, 65). Several cytokines, including TNF-α, have been implicated in the activation and execution of the autoimmune process (**Figure 3**, middle panel) (14, 66). Elevated levels of TNF-α have been observed in the CSF of MS patients, and these levels correlate with the severity and progression of the disease (67). Indeed, single nucleotide polymorphisms (SNP), encoding the TNFR1 gene (TNFRSF1A) have been linked to an elevated risk of developing MS (68). Moreover, mutations in the TNFR1 gene can lead to a condition known as TNF-receptor-associated periodic syndrome (TRAPS), characterized by recurrent episodes of fever, abdominal pain, and joint swelling in the absence of systemic autoimmunity (69). TRAPS can also manifest with neurological symptoms, referred to as CNS-TRAPS, which can bear resemblance to MS (70).

Similarly, TNF-α is upregulated in experimental autoimmune encephalomyelitis (EAE), the most commonly studied animal model of MS. Administration of TNF-α in these mice has shown to exacerbate the course of the disease (14, 71, 72). However, the role of TNF-α in the pathogenesis of MS and EAE is complex, and not all its effects are detrimental (14, 71). Contrary to expectations, studies have shown that TNF-α knockout mice develop equal or worse EAE compared to wild-type mice (73). Along the same lines, clinical studies of anti-TNF-α therapy in MS patients have been disappointing, with evidence of worsened disease activity, following treatment (14, 72, 73). One notable study was the lenercept trial, where MS patients receiving lenercept, a molecule designed to block TNF-α, experienced a higher frequency of relapses, earlier onset and worse neurological deficits compared to the placebo group (74).

Additionally, anti-TNF-α therapy, commonly used in the treatment of rheumatological disorders, has been associated with unexpected demyelinating events (75, 76). These incidents, along with the paradoxical worsening of MS upon TNF-α blockade, can be explained by the distinct and opposing functions of the TNFR1 and TNFR2 (14, 77). As mentioned earlier in this text, TNFR1 mediates pro-inflammatory signaling and can lead to cell apoptosis, while TNFR2 modulates immune functions and tissue preservation (3, 15). Specifically, TNFR2 is involved in Treg activation, which has a protective role against CNS autoimmunity. Furthermore, TNFR2 promotes oligodendrocyte differentiation and myelin repair (14, 24). More recent studies support the functional dichotomy of these receptors, and have shown that selective blockade of TNFR1 improves EAE outcomes, whereas knock out of TNFR2 worsens EAE disease (78–80). This has led to further investigations in developing therapeutic targets that can selectively target these receptors (80, 81).

## Neuro-Behçet’s disease

Behçet’s disease is a systemic inflammatory vasculitis of unknown origin (47). The disease is typically characterized by recurrent oral and genital ulcers, although it can involve other organs such as the heart, gastrointestinal track, kidneys and nervous system (82). Neurological complications, known as Neuro-Behçet’s, occur in approximately 10% of all cases and are considered on the most serious forms of the disease (83). CNS involvement can manifest as cerebral, brainstem, ocular or spinal cord symptoms, as well as cerebral sinus venous thrombosis, acute meningeal syndrome and stroke (83, 84).

The etiology and pathogenesis of Behçet’s disease are not completely understood and are likely multifactorial (85). Traditionally, an autoimmune etiology has been considered due to aberrant responses of the T and B cells of the adaptive immune system. However, the absence of disease- or antigen-specific T cell or antibody responses during the inflammatory episodes has led to the consideration of an autoinflammatory etiology related to dysfunction of the innate immune system (86). Another proposed etiology is a major histocompatibility complex (MHC) I -opathy, given the important association of the disease with HLA-B*51 allele (87). This allele is frequent among the populations of the “Silk Road”, where Behçet’s disease is most prevalent, although it can also be found in unaffected demographic groups (88). Environmental and epigenetic factors have also been implicated, and abnormal oral flora or herpes simplex 1 infection have been suggested as potential triggers and to play a relevant role in the disease (85, 87, 89).

The lack of a definitive etiology only emphasizes the complexity of this disease, a fact that also applies to its immunepathogenesis (85, 88). In Behçet’s disease, both the innate and the adaptive immune systems appear to be activated. NK cells, γδ T cells and neutrophils play a critical role in the disease, alongside Th1/Th2/T17 dysregulation and a decrease in T reg cells (82, 88) Cytokine studies have demonstrated elevated levels of TNF-α in Behçet’s disease, with a positive correlation between TNF-α levels and disease activity (82). There is also upregulated expression of TNFR1 and NF-κB in immune system cells. Interestingly, mutations in the TNFAIP3 gene, which encodes the regulatory protein A20 (a potent inhibitor of the NF-κB signaling pathway), have been associated with increased risk of developing Behçet’s disease and the lack of gene function results in Behçet’s disease-like pathological features (90, 91).

In Behçet’s, TNF-α is produced by macrophages, NK cells, T helper (Th)1 cells, Th22 cells, endothelial cells, neutrophils, and monocytes. Its production is triggered by enhanced signaling of Toll-like receptors (TLRs) (**Figure 3**, right panel). Under normal circumstances, TLRs are activated by endogenous and exogenous danger signals or damage-associated pattern molecules (DAMPs) (92). TLR stimulation results in NF-κB activation with the production of several pro-inflammatory cytokines, including TNF-α. The latter further augments this pro-inflammatory response by activating TNFR1signaling and the NF-κB pathway. Additionally, TNFR1 stimulation leads to apoptosis by activating caspase 8 (82).

Given its pivotal role, TNF-α has become one of the main therapeutic targets for Behçet’s disease (82, 85, 88). However, due to the rare incidence of the disease, clinical trial data on anti-TNF-α therapy are still limited (93). Nevertheless, most retrospective studies demonstrate a significant beneficial effect in treating uveitis, intestinal, vascular and neuro-Behcet’s symptoms (82, 89). Reportedly, 80% of neuro-Behçet’s patients have shown clinical improvement with such therapy, particularly with infliximab and adalimumab (89, 94). Although less commonly used, etanercept has also demonstrated clinical efficacy (95). Currently, there are no clinical trials comparing the efficacy and safety of the different anti-TNF therapies in Behçet’s disease.

## TNF-α blockage as a therapeutic target in CNS neuroinflammation

The most compelling evidence of the role of TNF-α in neuroinflammation and CNS diseases is provided by experimental and clinical studies on therapeutic blockade of this cytokine (13, 39, 82). Anti-TNF-α therapies, which are monoclonal antibodies targeting TNF-α, have been extensively studies. The ones used in CNS inflammatory diseases are summarized in **Table 1** (96). These therapies differ in terms of route of administration, dosing frequency, molecular structure, half-life and immunogenicity (53, 96). Among them, infliximab, a chimeric anti-TNF-α monoclonal antibody, is the most popular and commonly used in CNS inflammatory disorders. However, its chimeric molecule structure may lead to the development of neutralizing antibodies, reducing efficacy and increasing the risk of adverse reactions and paradoxical treatment responses (97, 98). Another commonly used anti-TNF-α therapy is adalimumab, which is similar to infliximab with the different of it being a fully humanized monoclonal antibody (54). Etanercept, on the other hand, consists of two copies of the TNFR2 fused to the human Fc portion of IgG (93).

**Table 1:** Anti-TNF-α drugs and its indications in CNS autoimmune disorders.

Drug*	Route	Half-life	Type of antibody	Molecule target	Structural differences	Indications in CNS disorders
Infliximab Remicade®	IV	7-12 d	Chimeric monoclonal IgG1 ab	Binds to monomeric^1^ and trimeric forms of soluble TNF-α and transmembrane TNF-α	Form stable complexes with TNF-α. Fix complement causing cell lyses of TNF-α expressing cells.	Neurosarcoidosis off-label, 1^st^ line. Behçet’s disease off-label
Adalimumab Humira®	SQ	10-20 d	Recombinant monoclonal IgG1 ab	Binds to monomeric^1^ and trimeric forms of soluble TNF-α and transmembrane TNF-α.		Neurosarcoidosis off-label, 2^nd^ line. Behçet’s disease off-label
Etanercept Embrel®	SQ	3 d	TNFR linked to the Fc portion of human IgG1	Binds primarily to the trimeric form of soluble TNF-α. Can also bind to transmembrane TNF-α	Forms unstable complexes with TNF-α. Doesn’t fix complement.	Behçet’s disease off-label
Atrosimab	Currently under development		Monoclonal humanized IgG1 ab	Binds to TNFR1 exclusively	Not applicable	Not applicable
TNC–scTNFR2^2^	Currently under development		Soluble, human TNFR2 agonist	TNFR2 agonist	Not applicable	Not applicable

* Two additional anti-TNF-α therapies, certolizumab and golimumab, approved by the Food and Drug Administration, are not included in this table. At present, there is very limited data on their use in CNS neuroinflammatory disorders.

^1^The monomeric form is the inactive form of TNF-α, whereas the trimeric form is the active form of TNF-α.

^2^Molecule engineered by genetic fusion of the trimerization domain of tenascin C to a TNFR2-selective single-chain TNF molecule, which is comprised of three TNF domains connected by short peptide linkers.

IV, intravenous; SQ, subcutaneous; d, days; TNFR, TNF-α receptor; CNS, central nervous system.

The main pharmacological effect of anti-TNF-α therapies is the neutralization of sTNF-α. Another important mechanism of action is antibody-dependent cellular cytotoxicity (ADCC) (99, 100). When these therapies bind to tmTNF-α on target cells, the Fc receptor interacts with a leukocyte, typically a NK cell, resulting in the lysis of the target cell (101). In addition, infliximab and adalimumab can induce complement dependent cytotoxicity (CDC) by activating the complement system, leading to lysis of certain cells expressing TNF-α on their membranes (56, 99, 102). Furthermore, both drugs have shown effects on cell cycle arrest and apoptosis through tmTNF-α, triggering outside-to-inside signals (reverse signaling) (99, 103, 104). In contrast, Etanercept lacks CDC activity and reverse signaling. These differences in the mechanism of action may explain why etanercept is not as effective as infliximab and adalimumab in eliminating TNF-producing cells and treating granulomatous diseases. However, it may also explain the lower risk of tuberculosis reactivation and granulomatous infections associated with etanercept (99, 105).

The unintended outcomes and the failed MS trials with anti-TNF-α therapies could be explained by the pleiotropic effects of TNF-α and the different downstream effects of TNFR1 and TNFR2 blockage. Understanding the positive impact of TNFR2 on oligodendrocyte homeostasis and remyelination has led to the development of selective TNFR blockage therapies (80, 81). One such example is atrosimab, an improved version of Atrosab, which has shown promising results in EAE (80). Reinforcing the concept of selective blockage, a recent study demonstrated that sequential use of a TNFR2 agonist (EHD2-scTNFR2) followed by atrosimab improved EAE outcomes (106). Although still being explored, the selective targeting of TNFR1 or TNFR2 presents new therapeutic prospects for the treatment of neuroinflammatory conditions, specifically MS. This approach may be able to minimize potential adverse effects associated with non-specific TNF-α blockade.

## Conclusion

TNF-α is one of the most widely studied cytokines. It activates different signaling pathways with diverse cellular effects, ranging from cell proliferation and synaptic plasticity to neuron cell death and neuroinflammation. The pleiotropism of TNF-α and its various roles in the CNS, whether homeostatic or deleterious, only emphasizes the functional complexity of this cytokine. Recognizing its master role in acute and chronic inflammation has spurred the development of a new class of biologic therapeutics. Anti-TNF-α therapy has proven to be effective in treating various autoimmune inflammatory diseases and has become a commonly used treatment approach for CNS autoimmune diseases. Additionally, newer and more specific therapeutics with selective blockage are under development, offering potential advancements in targeting specific aspects of TNF-α for therapeutic purposes. Future studies will be paramount in fully understanding the spectrum of TNF-α functions in the CNS and expanding the clinical applications of anti-TNF-α therapy.

## Author contributions

The author confirms being the sole contributor of this work and has approved it for publication.
